# Visualization of the Meridian System Based on Biomedical Information about Acupuncture Treatment

**DOI:** 10.1155/2013/872142

**Published:** 2013-05-28

**Authors:** In-Seon Lee, Soon-Ho Lee, Song-Yi Kim, Hyejung Lee, Hi-Joon Park, Younbyoung Chae

**Affiliations:** Acupuncture and Meridian Science Research Center, College of Korean Medicine, Kyung Hee University, 1 Hoegi-dong, Dongdaemun-gu, Seoul 130-701, Republic of Korea

## Abstract

The origin of the concept of the meridian system is closely connected with the treatment effects of acupuncture, and it serves as an empirical reference system in the clinical setting. Understanding the meridian channels would be a first step in enhancing the clinical efficacy of acupuncture treatment. To understand the relationship between the location of the disease and the sites of relevant acupoints, we investigated acupuncture treatment regimens for low-back pain in 37 clinical studies. We found that the most frequently used acupoints in the treatment of low-back pain were BL23 (51%), BL25 (43%), BL24 (32%), BL40 (32%), BL60 (32%), GB30 (32%), BL26 (28%), BL32 (28%), and GB34 (21%). For the example of low-back pain, we visualized the biomedical information (frequency rates) about acupuncture treatment on the meridians of a three-dimensional (3D) model of the human body. We found that both local and distal acupoints were used to treat low-back pain in clinical trials based on the meridian theory. We suggest a new model for the visualization of a data-driven 3D meridian system of biomedical information about the meridians and acupoints. These findings may be helpful in understanding the meridian system and revealing the effectiveness of acupuncture treatment.

## 1. Introduction

Acupuncture, which originated in East Asia, has been used as a therapeutic intervention for the treatment of various diseases and symptoms for more than 2500 years [1]. Despite cultural, historical, and sociopolitical differences, acupuncture is being used by practitioners in both Western and Eastern nations [2, 3]. Acupuncture treatment is based on a system of meridian channels through which vital energy, or Qi, flows [4]. The meridian system consists of twelve standard meridians, eight extraordinary meridians, and other collaterals. A classical text states, “*The twelve channels link the internal organs internally, and connect with the joints and limbs externally*,” suggesting that the body forms an organic whole by virtue of these internal and external connections and the upward and downward distribution of the meridian channels. A detailed understanding of the meridian system helps in understanding and contextualizing those connections and in developing a broad sense of acupoints and their actions.

Ancient acupuncture practitioners found therapeutic actions and influences of particular points in relation to particular internal organs, as well as to other parts of the body part far from the point. The observation of points along a certain line sharing similar indications led practitioners to categorize them based on the “lines” on which they were located. The meridian system shows constellations of acupoints that have common therapeutic effects in acupuncture for the specific symptoms of body parts, and it is used to explain the remote effects of acupuncture treatment. For example, the large intestine meridian, also traditionally called the dental meridian, is a line from the index finger to the mouth and nose, and stimulation of points along this line is known to be useful for treating dental pain and facial palsy [5, 6]. The meridian system has been considered to reflect a systematic body of empirical knowledge that functions as the basis for acupuncture treatment [7]. Understanding the essence of the meridian system helps us in developing an understanding of the interconnections that underlie pathology in a particular disease.

Because the meridian system has a large amount of information regarding the human body surface that is hard to classify by a literal description alone, the ancient people of China invented several methods to display information about acupoints and meridians on the human body surface. Historically, many pictures have been used to describe the meridian system, such as the well-known Mingtang Diagram. The *Illustrated Manual of Points for Acupuncture and Moxibustion on a Bronze Statue with Acupoints* (also called the *Illustrated Classic of Acupuncture Points of the Bronze Model*) and its accompanying bronze statue, developed in the Song Dynasty, defined the first standardized acupoints and constituted the official standardized model of the meridian system [8]. The 3D statue is usually made of bronze and it is still widely used in educational and clinical fields. The Mingtang Diagram and the bronze statue were gradually combined, with descriptions of the relationship between internal organs and the skeletal structure and meridian system [9]. In the West, Ten Rhyne first reported in person East Asian medical practices, including acupuncture, as a medical officer in 1683. He sought to create an interpretative synthesis between Chinese and Western medicine, using the pictures in an attempt at “translation” [10]. 

Ancient East Asian people intended to explain empirical knowledge using the ancient infographics of the meridian system, but these were limited in reflecting theoretical or abstract meanings [11]. Indeed, the illustrations of the meridian system and acupoints may have made people misunderstand the essential meaning of the meridian system as actual substantial channels. Over the last few decades, many studies have suggested distinctive biophysical features of acupoints and the meridian system, such as high electrical conductance [12–14], nitric oxide levels [9, 15], acupuncture sensation patterns [16], and possible relationships with connective tissue planes [17, 18]. Nevertheless, the scientific evidence on the biophysical existence of acupoints and meridians in humans still needs further explanation. The Bonghan system was originally proposed in the early 1960s by Bonghan Kim and has more recently been replicated by others [19]. However, the conjecture that primovessels (Bonghan ducts) serve the role of meridians in acupuncture has not yet been established. To understand the meridian system properly, we have to start from understanding the origin and clinical significance of the meridian system. The meridian system is a method that proposes connections among different areas, organs, and functions; thus, it is associated with specific patterns of disharmony.

The concept of meridians and acupoints can serve as an empirical reference system in the clinical setting regardless of the anatomical precision of the meridians and acupoints. The traditional Mingtang Diagram and bronze statue only depict information about the locations of acupoints and the meridians. When we want to know which acupoint(s) would be useful in treating a certain disease, we cannot find answers from these illustrations and/or this statue. Here, we suggest a new model of the meridian system that incorporates clinical data with the meridian map, an infographic expressing biomedical information about the meridian system and acupoints.

## 2. Methods

### 2.1. Selection of Biomedical Information

In the current study, we consider the example of acupuncture treatment regimens for low-back pain and visualize the meridian system in terms of biomedical information about acupuncture treatments. To determine which acupoints are useful in treating low-back pain, we extracted acupuncture-point-related information from clinical acupuncture treatment trials for low-back pain.

### 2.2. Selection of Studies

We searched Medline, five Korean databases, relevant journals, and trial registries for randomized, controlled trials of acupuncture that involved needling for low-back pain. Reference lists of all the papers located and relevant reviews were checked for missing articles. Search terms used for Medline were as follows: (((acupuncture [Title/Abstract] AND low-back pain [Title/Abstract]) OR (acupuncture [Title/Abstract] AND lumbago [Title/Abstract])) OR (acupuncture [Title/Abstract] AND lumbar pain [Title/Abstract])) OR (acupuncture [Title/Abstract] AND lower back pain [Title/Abstract]), with slight modifications for individual searches in each database, over the time period from January 1980 to April 2012.

We included randomized controlled trials, clinical controlled trials, and case reports/series that used needle-type acupuncture (manual acupuncture, electronic acupuncture) to treat low-back pain written in English or Korean. To find general acupuncture treatment regimens for low-back pain, we investigated the frequency of the acupoints selected in each study among the trials. We reanalyzed data from previous published study [20] using data-based meridian map technique, designed to both estimate the magnitude of the resulting frequency values and provide interpretable maps of which acupoints are useful to treat low-back pain. Detailed information was provided in our previous systematic review on the selection of acupoints for low-back pain [20].

### 2.3. Visualization of the Meridian Map

The algorithm aims to identify acupoints that are related to a convergence of use across a series of studies. The frequency of acupoint use (%) was calculated as the number of studies using a certain acupoint in each study divided by the total number of studies × 100. The meridian systems were overlaid on the “3D Meridian map” anatomical template using Autodesk 3ds Max Design 2009 software. The basic anatomical template was TurboSquid's 3D human male body (http://www.turbosquid.com). The acupoints data were labeled according to the magnitude of the resulting frequency values.

## 3. Results

### 3.1. Most Frequently Used Acupoints for Low-Back Pain

**Figure 1:**
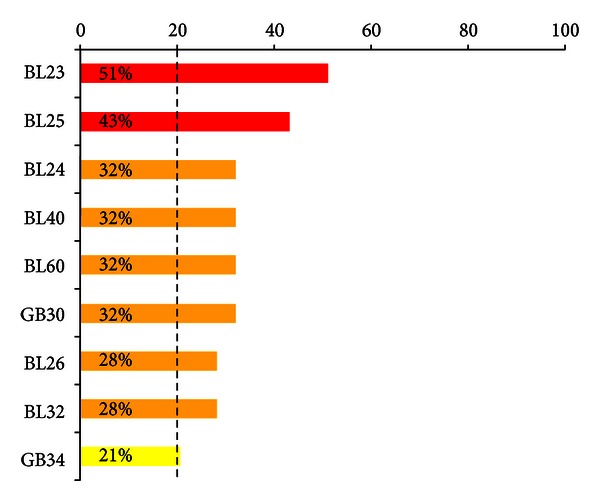
Frequency of acupoints in 53 studies. Frequency of acupoints selected in each study (%). Frequency % = number of studies using certain acupoint/total number of studies × 100. The most frequently adopted acupoints for the treatment of the low-back pain (used in >20% of the studies) were BL23 (51%), BL25 (43%), BL24 (32%), BL40 (32%), BL60 (32%), GB30 (32%), BL26 (28%), BL32 (28%), and GB34 (21%).

Through this systematic review, we found 37 articles (17 Korean, 20 international) and 53 studies (28 Korean, 25 international). The most frequently used acupoints for the treatment of the low-back pain (used in over 20% of the 53 studies) were BL23 (51%), BL25 (43%), BL24 (32%), BL40 (32%), BL60 (32%), GB30 (32%), BL26 (28%), BL32 (28%), and GB34 (21%) (Figure 1).

### 3.2. Visualization of Data-Driven 3D Meridian System for Low-Back Pain

**Figure 2:**
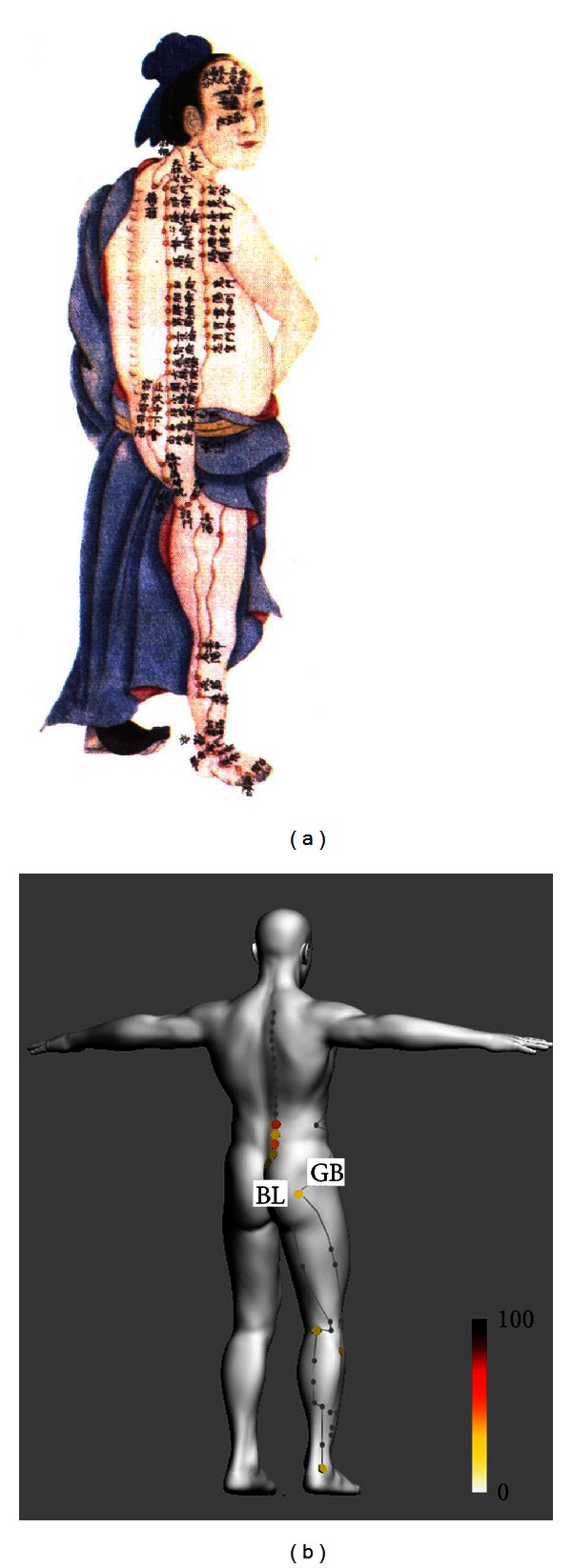
(a) A traditional Mingtang Diagram for the bladder meridian from the Qing Dynasty. (b) A new model of a data-driven 3D visualization of the meridian system based on biomedical information on the meridians and acupoints for low-back pain. Acupoints are marked according to the frequency of their use in the treatment of low-back pain as reported in the clinical data.

We demonstrated a data-driven 3D meridian system for low-back pain based on frequency analysis of the results of a systematic review of the relevant literature.  Figure 2(b) shows these acupoints according to frequency of use (>20% of the studies) for low-back pain treatment. The acupoints include seven acupoints (BL23, BL24, BL25, BL26, and BL32 as local points and BL40 and BL60 as distal points) on the *bladder meridian* and two acupoints (GB30 as a local point and GB34 as a distal point) on the *gallbladder meridian*.

## 4. Discussion

Using the example of low-back pain, we suggest a new model of the meridian system based on biomedical information about the meridians and acupoints. In the current study, we found that the most frequently used acupoints for the treatment of low-back pain in 53 clinical studies were BL23 (51%), BL25 (43%), BL24 (32%), BL40 (32%), BL60 (32%), GB30 (32%), BL26 (28%), BL32 (28%), and GB34 (21%; Figure 1). The traditional Mingtang Diagram was regarded as explaining empirical knowledge using the ancient infographics of the meridian system; for example, it indicated that some acupoints on the lower limb could exert remote control of the posterior part of the body, such as the back, neck and head (i.e., the bladder meridian; Figure 2(a)). However, these illustrations do not deal with biomedical information based on clinical data; instead, they express theoretical or abstract meanings of the meridian system. Here, we propose that the acupoints can be depicted according to the frequency of their use in the treatment of low-back pain based on a comprehensive set of quantitative clinical data (Figure 2(b)). This should be helpful in understanding the essential meaning of the meridian system and in providing clinical guideline, evaluating the results of clinical trials appropriately and assessing the effectiveness of the acupoints used. Data-based meridian map techniques estimate the magnitude of the resulting frequency values and provide interpretable maps of which acupoints are useful to treat low-back pain.

The current study showed which acupoints were generally used in treating low-back pain based on 53 treatment regimens in clinical trials. The analysis of these regimes showed that the bladder meridian (BL23, BL24, BL25, BL26, BL32, BL40, and BL60) and the gall bladder meridian (GB30, GB40) were most frequently used in treating low-back pain. These findings were considerably consistent with the traditional Chinese medicine theory, in which some acupoints, including BL23, BL25, GV3, BL40, GB30, are commonly used in the treatment of chronic low-back pain [21]. As the symptoms of low-back pain were frequently located in the low-back area, the characteristics of the bladder meridian would suggest prominent connections between the disease area and the foci of acupoints. Both local (BL23, BL24, BL25, BL26, and BL32) and distal (BL40, BL60) acupoints based on meridian theory were used to treat low-back pain in these clinical trials. The observation that points along the posterior line in the low back and the posterior line in the lower limb share similar indications could be explained in terms of the bladder meridian on which they are located. Biomedical information about the meridian system could explain how the concepts of the meridians and acupoints served as an empirical reference system in the clinical setting.

In a German study, after standardized acupuncture (plus some additional points) for 6 months, the response rate was 47.6% in the real acupuncture group, 44.2% in the sham acupuncture group, and 27.4% in the conventional therapy group [22]. A meta-analysis in 2008, which involved 23 trials (*n* = 6359), demonstrated that acupuncture was more effective than no treatment but that real acupuncture treatments were not more effective than sham acupuncture treatment [23]. However, because extensive clinical trials have suggested that acupuncture may be more effective than usual care, it may best be understood as a useful supplement to other forms of conventional therapy for nonspecific low-back pain [21]. Treatment regimens of acupuncture for low-back pain can differ according to the type of reference source, treatment frequency, the points chosen, the number of points needled per session, the duration and number of sessions, and cointerventions [24]. Because the selection of acupoints is highly associated with the outcome of acupuncture treatment, it is important to evaluate the role of each acupoint in clinical trials. As most of clinical trials only provide the overall clinical efficacy of acupuncture treatment and do not deal with issues related to acupoint, it would be very difficult to determine which acupoints were most useful in treating low-back pain from reports of the previous clinical trials.

The present study visualized biomedical information (frequency-based information) on the meridians of 3D surface anatomical models of the human body for low-back pain. These tools could be extended to other kinds of diseases with more meaningful biomedical information (efficacy-based information and/or bibliographical information). The computer modeling of biological systems has been considered an important technique for organizing and integrating vast amounts of biological information [25]. Systems biology has been developed to achieve the goal of understanding the interactions in complex pathways of multiple components and between multiple levels of function using mathematics and computing [26]. In the near future, these computational approaches to the meridian system will become more integrated and will be more readily linked with bioinformatic databases from clinical trials and/or classical texts. At that point, it will be possible to gather a better-organized data-based 3D visualized meridian system containing the therapeutic effects of acupuncture. Collection of further meaningful biomedical information on acupuncture with a multiscale modeling framework in a variety of diseases is still needed.

In sum, we suggest a new model for the visualization of a data-driven 3D meridian system, “*The Meridian Map*,” which expresses available biomedical information on the meridian system and acupoints using infographic means. The significance of the meridian system is not based simply on the twelve lines of the whole organism but also relies on the logic of the interconnections between particular diseases and acupuncture sites. Understanding the essence of the meridian channels enables people to grasp the principles of the selection of acupoints and to enhance the clinical efficacy of acupuncture treatment in various diseases.
